# P4B: A novel probe to study cellulose synthesis and microtubule dynamics

**DOI:** 10.1093/plphys/kiae305

**Published:** 2024-05-24

**Authors:** Nicola Trozzi

**Affiliations:** Assistant Features Editor, Plant Physiology, American Society of Plant Biologists; John Innes Centre, Norwich Research Park, Norwich, NR4 7UH, UK; Department of Plant Molecular Biology, University of Lausanne, CH-1015 Lausanne, Switzerland

Cellulose is the most abundant biopolymer on Earth and a major component of plant cell walls. In growing cells, cellulose is synthesized at the plasma membrane by cellulose synthase complexes (CSCs), which are assembled in the Golgi apparatus and trafficked to the cell surface (McFarlane et al. 2014). Despite the importance of cellulose in plant growth and development, many aspects of its synthesis remain unknown. Chemical genetics has proven to be a powerful approach to study complex cellular processes like cellulose biosynthesis (Brabham and DeBolt 2013). By screening chemical libraries for compounds that affect plant growth, researchers can identify new cellulose biosynthesis inhibitors (CBIs) and gain insights into the molecular mechanisms of cellulose production.

In this issue of *Plant Physiology*, Renou et al. (2024) report the identification and characterization of a novel CBI named P4B (2-phenyl-1-[4-(6-(piperidin-1-yl) pyridazin-3-yl) piperazin-1-yl] butan-1-one), which exhibits a unique mode of action compared with previously known CBIs (Renou et al. 2024). Through a chemical genetics screen in *Arabidopsis thaliana*, the authors discovered that P4B strongly inhibits seedling growth. Further analyses revealed that P4B reduces crystalline cellulose content by 40% to 50%, comparable with the well-known CBI isoxaben (Tateno et al. 2016). The researchers also identified a mutant, *cesa^3pbr1^*, which is resistant to the growth inhibition and cellulose reduction caused by P4B. This mutation affects the *CESA3*, which encodes a catalytic subunit of the CSC (Desprez et al. 2007), suggesting *CESA3* may be a direct or indirect target of P4B.

To investigate how P4B affects cellulose synthesis, Renou et al. used spinning disk confocal microscopy to track fluorescently tagged CSCs in living cells treated with the inhibitor. Surprisingly, short-term P4B treatment did not stop the movement of CSCs in the plasma membrane, which is driven by the polymerization of cellulose (Paredez et al. 2006). This contrasts with the effects of many other CBIs that clear the CSCs from the cell surface (Crowell et al. 2009; Gutierrez et al. 2009). Instead, P4B reduced the delivery rate of CSCs to the plasma membrane, leading to a decrease in their density at the cell surface without causing accumulation in Golgi-derived vesicles, as seen with some other CBIs (Fig.). This unique effect of P4B on CSC secretion, without causing accumulation in Golgi-derived vesicles, sets it apart from most known CBIs and suggests a novel mechanism of action. The *cesa3^pbr1^* mutant was not affected in this delivery process, highlighting the importance of *CESA3* in P4B's mode of action. However, it is important to note that while the *cesa3^pbr1^* mutation suggests a link between *CESA3* and P4B, there is no direct evidence of physical interaction between the inhibitor and the protein.

**Figure.**
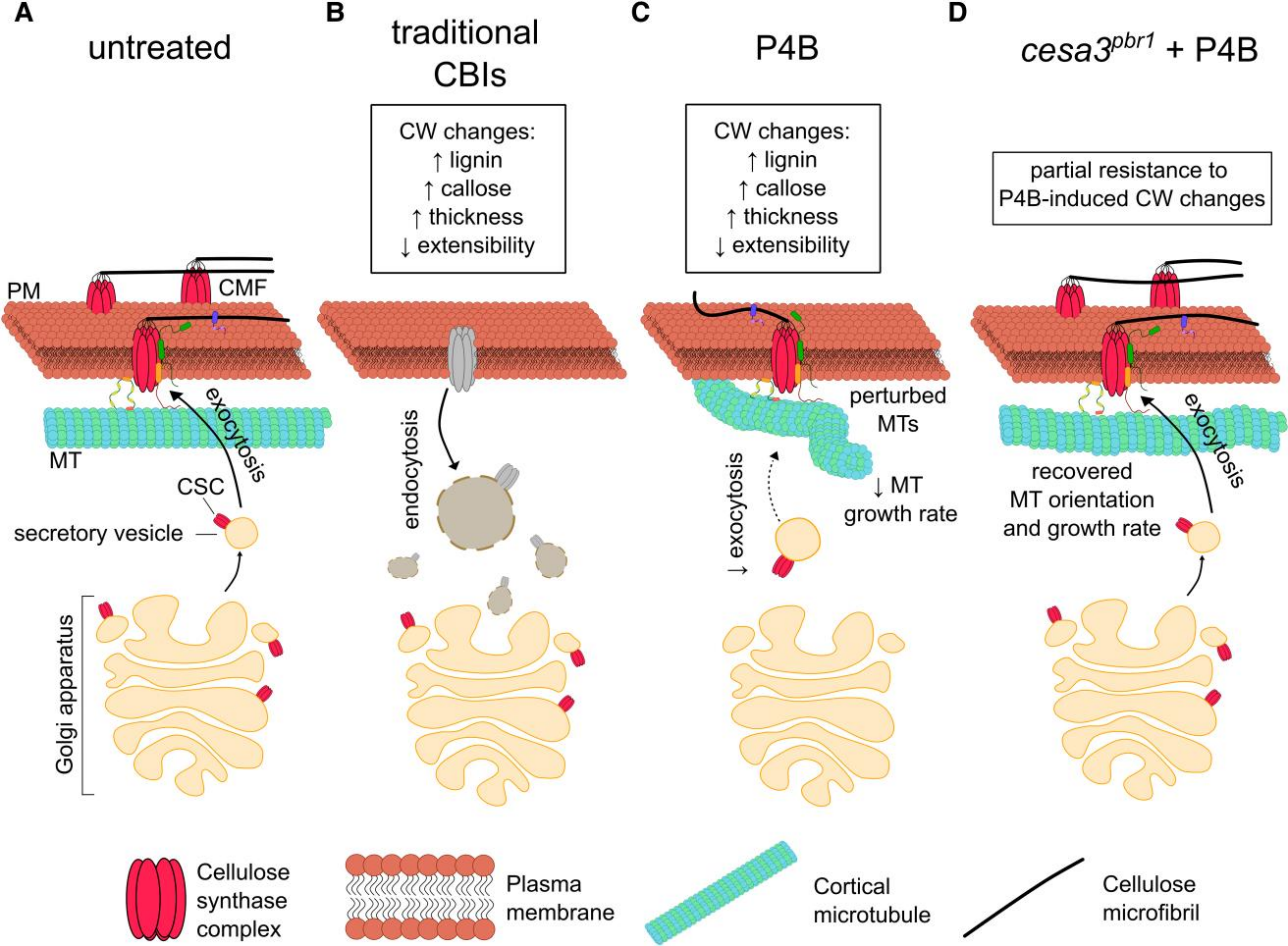
Schematic representation of the effects of P4B and other CBIs on CSC trafficking and microtubule dynamics. **A)** In untreated cells, CSCs are synthesized in the Golgi apparatus, secreted to the plasma membrane, and move along cortical microtubules while synthesizing cellulose. **B)** Traditional CBIs cause CSCs to be cleared from the plasma membrane and accumulate in Golgi-derived vesicles. **C)** P4B decreases CSC secretion to the plasma membrane without causing accumulation in vesicles and also perturbs microtubule orientation and dynamics. **D)** The *cesa3^pbr1^* mutant is resistant to the effects of P4B on CSC secretion and partially resistant to the effects on microtubules and cell wall composition.

Interestingly, P4B treatment also altered the behavior of cortical microtubules, which guide the trajectories of CSCs in the plasma membrane (Bringmann et al. 2012). Within a few hours, P4B caused the microtubules to reorient from a transverse to oblique alignment and decreased their growth rates at the plus ends. These results suggest P4B perturbs microtubule dynamics in addition to affecting CSC trafficking. The ability of P4B to alter microtubule dynamics is another distinctive feature of this inhibitor, as this effect is not commonly observed with other CBIs.

Longer P4B treatments mimicked some effects seen with other CBIs, such as decreased CSC movement, ectopic lignin and callose deposition, and an abnormal wall architecture that could not expand properly (Caño-Delgado et al. 2003; Tateno et al. 2016). The *cesa3^pbr1^* mutant was resistant to many of these effects, reinforcing the link between *CESA3* and P4B. However, the mutant still showed some PB4-induced changes in CSC velocity and wall composition not seen with short-term treatments, implying that P4B may have additional targets that require longer to respond.

In summary, Renou et al. identified P4B as a potent new inhibitor of cellulose synthesis that mainly acts by decreasing CSC secretion to the plasma membrane. This mode of action, which also alters microtubule dynamics, differs from many known CBIs and provides a new tool to dissect the complex process of cellulose synthesis in plants. While the *cesa3* mutations confer resistance to P4B, implying a role for the CESA3 subunit in regulating CSC trafficking and microtubule dynamics, further studies are needed to determine if there is a direct interaction between P4B and *CESA3*. The ability of P4B to alter microtubule dynamics and CSC trafficking highlights its potential as a valuable tool to study the broader effects on these processes, in addition to its impact on cellulose synthesis. Future work could focus on how P4B interacts with *CESA3* and other components of the CSC to control its assembly and delivery to the cell surface. The link between P4B, *CESA3*, and microtubule behavior also warrants further investigation, as it may reveal new aspects of the cross-talk between the cell wall and cytoskeleton. As our understanding of cellulose synthesis continues to grow, small molecules like P4B will undoubtedly play a central role in explaining the mechanisms of this essential process in plant biology.
